# Association between metabolic obesity phenotype, transition of metabolic phenotypes and the risk of hyperuricemia in Chinese adults: A cohort study

**DOI:** 10.1097/MD.0000000000032094

**Published:** 2022-11-25

**Authors:** Wenjing Zhao, Cheng Zhao

**Affiliations:** a Dezhou Center for Disease Control and Prevention, Dezhou, PR China.

**Keywords:** hyperuricemia, metabolism, obesity, risk factors

## Abstract

Prospective evidence on the association of obesity and metabolic health status and its transition over time with the risk of hyperuricemia in the Chinese population is limited. This study aims to investigate the phenotypic transition characteristics of metabolic obesity in Chinese adults and its association with hyperuricemia. Using the China Health and Retirement Longitudinal Survey (CHARLS) survey data in 2011 and 2015, 6059 adults aged ≥ 18 years were selected as the research people. The participants’ general information, living habits, blood sample testing, and blood uric acid testing data during follow-up were extracted. According to body weight and metabolic health status, obesity phenotypes were divided into: metabolically normal weight group (MHNW), metabolically normal overweight/obesity group (MHOWO); metabolically abnormal normal weight group (MUNW); metabolically abnormal overweight/obese group (MUHOWO). Multiple linear regression was used to evaluate the correlation between metabolic obesity phenotype and serum uric acid level, and logistic regression model was used to analyze the association of metabolic obesity phenotype and transition with the risk of hyperuricemia. The average age of all subjects was (58.62 ± 8.93) years old, and 42.1% were male. The MHOWO phenotype was present in 19.2% of the general population and 48.6% of the baseline who were overweight or obese population. During the 4-year follow-up period, only 10.7% of participants with MHNW at baseline converted to MHOWO. Among MHOWO participants, 21.2% converted to MUHOWO. MHOWO also increased the risk of hyperuricemia (OR, 1.57; 95% CI 1.15–2.13; *P* = .004), both in obese and normal-weight individuals, even when metabolic status changed from unhealthy to healthy. Risk of hyperuricemia was high among those who remained metabolically unhealthy but of normal weight (OR, 3.09; 95% CI 1.51–6.30; *P *= .001). MHOWO also increases the risk of hyperuricemia, and MHOWO remains stable or changes to MUHOWO, which increases the risk of hyperuricemia. Therefore, close attention should be paid to the transition of metabolic health status over time, and individualized prevention strategies should be focused on metabolically unhealthy and obese individuals.

## 1. Introduction

Hyperuricemia is a metabolic disease characterized by abnormally elevated blood uric acid levels. It not only directly leads to gout and impaired renal function, but is also closely related to various chronic diseases such as diabetes, hypertension, and cardiovascular and cerebrovascular diseases. In 2010, the prevalence of hyperuricemia among Chinese adults was 8.4%.^[1]^ In 2015, a survey of adults aged 18-59 years in 15 provinces across the country found that the prevalence of hyperuricemia was 9.8%.^[2]^ The “Guidelines for Diagnosis and Treatment of Hyperuricemia and Gout in China (2019 Edition)” stated that the prevalence of hyperuricemia in China has reached 13.3%.^[3]^ Hyperuricemia has posed a serious threat to the health of Chinese residents.

Obesity and its associated metabolic disorders are major risk factors for hyperuricemia globally. However, there are differences in metabolic factors among obese patients, and some obese patients do not develop metabolic disorders and are defined as metabolically healthy obesity (MHOWO), but whether MHOWO is a healthy state is still controversial.^[4,5]^ However, the MHOWO phenotype is not a stable state,^[6]^ and studies have shown that 33% to 52% of MHOWO patients will transform into a metabolically unhealthy phenotype.^[7,8]^ However, there is no evidence on how MHOWO transitions in the Chinese population and its association with the risk of hyperuricemia. It remains unclear how metabolic factors change over time across and how such dynamic metabolic transitions affects hyperuricemia risk among Chinese adults. Studies assessing the hyperuricemia hazards of dynamic metabolic transitions over time in China, are of great both public health and clinical significance and provide strategies for early intervention.

In this study, the data from the China Health and Retirement Survey (CHARLS), a nationwide prospective cohort study, were used to analyze the change of metabolic obesity phenotype during the follow-up period and its association with the risk of hyperuricemia, so as to provide a scientific basis for the intervention and prevention of related high-risk populations.

## 2. Materials and methods

### 2.1. Data sources

This study used data from the China Health and Retirement Longitudinal Study (CHARLS), a longitudinal, nationally representative study of middle-aged and elderly residents (≥45 years old) in China. The research survey draws samples according to a multi-stage regional probability sampling design, and constructs a high-quality public database containing extensive Chinese population data. The database contains detailed information on socioeconomic and demographic factors, household information, and health status. The original sample consisted of 17,708 respondents randomly selected from 450 villages/neighborhood committees in 150 counties/districts in 28 provinces in 2011. Face-to-face computer-assisted personal interviews were conducted with respondents every 2 years. The CHARLS study was approved by the Biomedical Ethics Committee of Peking University (IRB00001052-11015), and all subjects participating in the study signed the informed consent^.[7]^

This study selected 2011 as the baseline and 6059 adult residents with complete blood uric acid measurement data and demographic data in 2 follow-up surveys in 2015.

### 2.2. Selection of research subjects

The inclusion criteria are: Those who participated in 2 surveys in 2011 and 2015 at the same time; Complete blood test and physical examination data.

The exclusion criteria were: Patients with hyperuricemia at baseline examination; Missing key variables such as height, weight, blood pressure, and blood sugar.

### 2.3. Research methods

(1) Extract relevant information (age, gender, education level, marital status), living habits (current smoking status, drinking status), body mass index (BMI), hypertension, blood lipid profile, serum uric acid from the CHARLS database value. Four subgroups were divided according to the metabolic obesity phenotype.
(2) Relevant definitions
  ① Hyperuricemia: According to the definition of “Chinese Guidelines for Diagnosis and Treatment of Hyperuricemia and Gout (2019 Edition),” hyperuricemia is determined when serum uric acid exceeds 420 μmol/L (7 mg/dL).
  ② Obesity: According to the recommendations of the China Obesity Working Group, a BMI value ≥ 24 kg/m^2^ is determined as overweight; a BMI ≥ 28 kg/m^2^ is considered obese.^[9]^
  ③ Metabolic syndrome: According to the “Chinese Guidelines for the Prevention and Treatment of Type 2 Diabetes (2020 Edition),” those with or exceeding the following 3 components are defined as metabolic syndrome.^[10]^
    a. Abdominal obesity: female waist circumference ≥ 85 cm, male waist circumference ≥ 90 cm;
    b. Hyperglycemia: fasting blood glucose ≥ 6.1 mmol/L or 2 hours post-glucose load blood glucose ≥ 7.8 mmol/L and (or) those who have been diagnosed with diabetes and treated;
    c. Hypertension: blood pressure ≥ 130/85 mm Hg and (or) confirmed hypertension and treatment;
    d. Fasting triglyceride ≥ 1.70 mmol/L;
    e. Fasting HDL-C < 1.04 mmol/L.
(4) Obesity phenotype: According to the weight status grouping and metabolic syndrome components, the research subjects were defined as 4 types of obesity phenotypes: metabolically healthy normal weight group (MHNW); metabolically normal overweight/obesity group (metabolically healthy overweight/obesity, MHOWO); metabolically unhealthy normal weight (MUNW); metabolically unhealthy overweight/obesity (MUHOWO) group.

### 2.4. Statistical methods

Continuous variables were expressed as mean ± standard deviation (𝑥̅ ± 𝑆), and categorical variables were expressed as frequencies and percentages (n, %). When the data of different groups obeyed the normal distribution and the variance was homogeneous, analysis of variance was used, and the chi-square test was used to compare the classification data of different groups. Multiple linear regression was used to evaluate the correlation between metabolic obesity phenotype and serum uric acid level, and logistic regression model was used to analyze the association of metabolic obesity phenotype and transition with the risk of hyperuricemia. All data analyses were performed in SPSS 22.0 with α = 0.05.

## 3. Results

A total of 6059 participants were included in this study according to the selection criteria, including 2664 males (42.1%) and 3395 females (57.9%). According to metabolic health and obesity status, 1770 (30.0%) participants were metabolically unhealthy and 2499 (39.5%) participants were overweight or obese. The MHOWO phenotype was present in 19.2% of the general population (n = 1214) and in 48.6% of the overweight and obese population. It is worth noting that there were statistically significant differences in education level, smoking and drinking, blood pressure, blood lipid profile and other indicators among the groups (*P* < .05), as shown in Table 1.

**Table 1:** Basic characteristics of investigators with different metabolic obesity phenotypes (n = 6059).

Variable		Normal weight		Overweight/Obesity			
N(%)		Metabolically healthy	Metabolically unhealthy	Metabolically healthy	Metabolically unhealthy	F/χ^2^	*P*
Age		59.33 ± 9.16	61.71 ± 9.07	55.74 ± 8.16	58.50 ± 8.33	70.13	<.001
Sex						147.20	<.001
	Male	1583(51.5)	189(39.0)	414(34.1)	478 (37.2)		
	Female	1492(48.5)	296(61.0)	800(65.9)	807(62.8)		
BMI (kg/m^2^)		20.99 ± 1.92	22.19 ± 1.49	26.56 ± 2.90	27.54 ± 2.68	3333.67	<.001
Education†						14.25	.002
Middle school		2836(92.2)	453(93.4)	1085(89.4)	1157(90.0)		
High school		239(7.8)	32 (6.6)	129(10.6)	128 (10.0)		
Marital status						60.46	<.001
Married		2709(88.1)	390(80.4)	1125(92.7)	1168(90.9)		
Non-married		366(11.9)	95 (19.6)	89(7.3)	117 (9.1)		
Smoking status						130.80	<.001
	Yes	1348(43.8)	177(36.5)	329(27.1)	401(31.2)		
	No	1727(56.2)	308 (63.5)	885(72.9)	884 (68.8)		
Drinking status‡						77.65	<.001
>1		866(28.2)	390(80.4)	250 (20.6)	234(18.2)		
<1		250(8.1)	95 (19.6)	96(7.9)	90 (7.0)		
Never		1959(63.7)	356(73.4)	868 (71.5)	961(74.8)		
Waistline (cm)		78.01 ± 9.24	84.93 ± 8.76	89.98 ± 9.56	95.45 ± 8.47	1285.00	<.001
Uric acid (mg/dL)		4.17 ± 1.05	4.42 ± 1.11	4.18 ± 1.02	4.53 ± 1.05	40.89	<.001
Triglycerides(mg/dL)		98.91 ± 52.68	212.09 ± 157.53	104.63 ± 42.73	225.91 ± 179.81	1746	<.001
Total cholesterol (mg/dL)		188.54 ± 35.52	199.15 ± 41.77	193.78 ± 36.58	201.12 ± 42.48	38.42	<.001
LDL (mg/dL)		113.75 ± 31.49	199.15 ± 41.77	123.02 ± 33.01	116.74 ± 38.87	23.86	<.001
HDL (mg/dL)		56.86 ± 15.10	40.12 ± 12.65	52.27 ± 11.60	40.62 ± 11.74	547.26	<.001
SBP (mm Hg)		124.40 ± 19.42	138.43 ± 21.25	127.44 ± 19.96	139.18 ± 20.14	205.09	<.001
DBP (mm Hg)		72.20 ± 11.25	78.11 ± 11.84	75.88 ± 11.61	81.34 ± 11.58	200.77	<.001

BMI = body mass index, DBP = diastolic blood pressure, HDL = high density lipoprotein, LDL = low density lipoprotein, SBP = systolic blood pressure.

† Education level is divided into middle school and below, and high school and above.

‡ The unit of drinking status is times/month.

**Figure 1.**
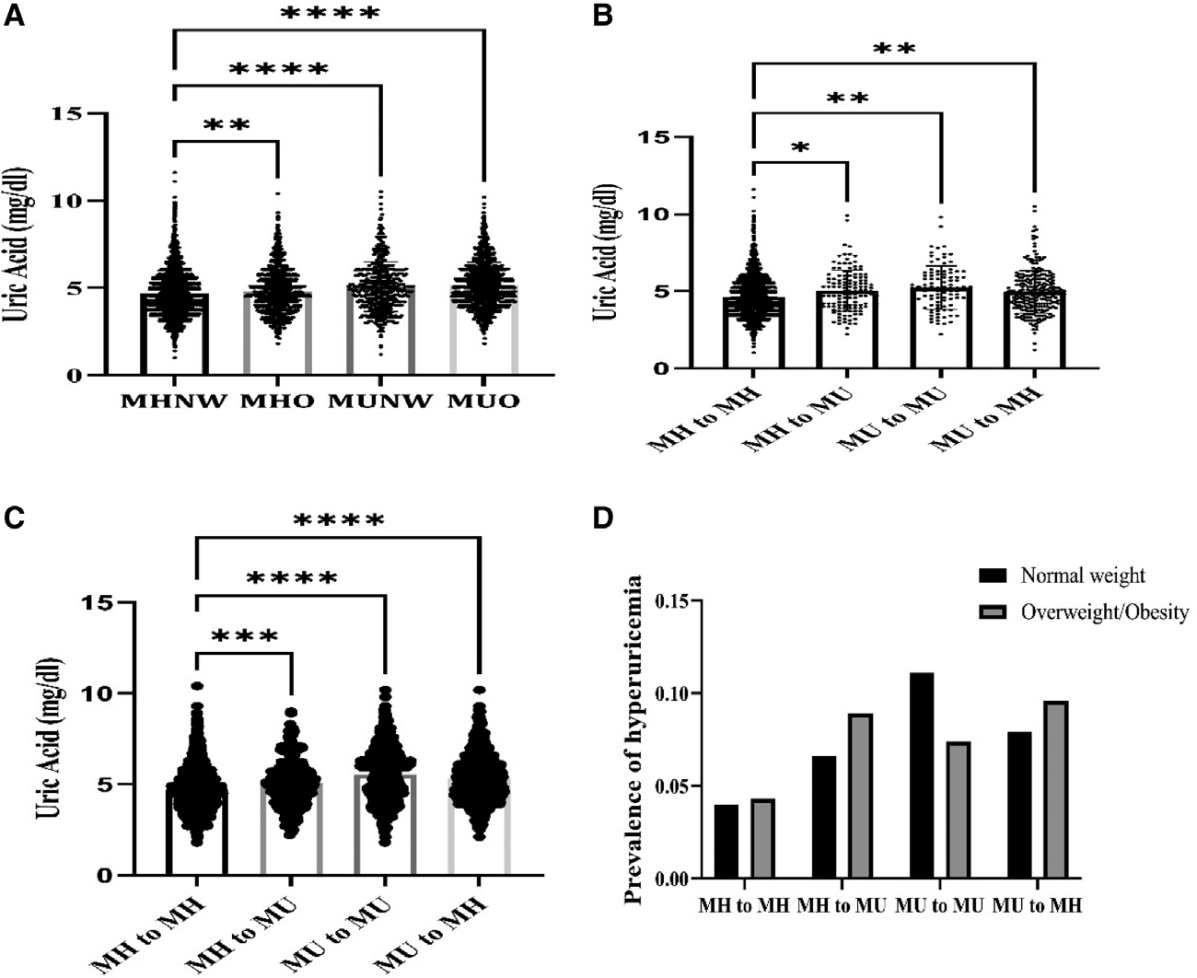
Comparison of serum uric acid value and prevalence of hyperuricemia in different groups. (A) Serum uric acid values between different phenotypes in 2015. (B) Serum uric acid values in different phenotypic transition groups in normal weight subjects in 2015. (C) Serum uric acid values in different phenotypic transition groups of overweight and obese individuals in 2015. (D) The prevalence of hyperuricemia in different phenotypic transition groups in 2015.

The follow-up data in 2015 showed that the mean ± standard deviation of serum uric acid values between different phenotypes were, MHNW: 4.69 ± 1.31 mg/dL, MHOWO: 4.80 ± 1.27 mg/dL, MUNW: 5.06 ± 1.42 mg/dL and MUHOWO: 5.16 ± 1.30 mg/dL. Using the Bonferroni method for multiple correction, pairwise comparison found that the serum uric acid value in the MHNW group was lower than that in the MHOWO, MUNW and MUHOWO groups, and the difference was statistically significant (*P* < .05), as shown in Figure 1A. The mean ± standard deviation of serum uric acid values ​​among the 4 phenotypic transitions in normal weight subjects were, respectively, MH to MH: 4.64 ± 1.28 mg/dL, MH to MU: 5.03 ± 1.33 mg/dL, MU to MU: 5.24 ± 1.41 mg/dL, MU to MH: 4.95 ± 1.39 mg/dL. The Bonferroni method was used for multiple correction, and it was found that the blood uric acid value of the MH to MH group was lower than that of the other 3 groups, and the difference was statistically significant (*P* < .05), as shown in Figure 1B. The mean ± standard deviation of serum uric acid values ​​among the 4 phenotypic transitions in overweight and obese individuals were, respectively, MH to MH: 4.71 ± 1.23 mg/dL, MH to MU: 5.05 ± 1.30 mg/dL, MU to MU: 5.54 ± 1.45 mg/dL, MU to MH: 5.30 ± 1.27 mg/dL. Using the Bonferroni method for multiple corrections, it was found that the blood uric acid value of the MH to MH group was lower than that of the other 3 groups in pairwise comparison, and the difference was statistically significant (*P* < .05), as shown in Figure 1C. The 3 phenotypic transition groups, MH to MU, MU to MU, and MU to MH, had a higher prevalence of hyperuricemia than the metabolically stable (MH to MH) group in both normal-weight and obese subjects, see Figure 1D.

During the 4-year follow-up period, there were 370 new cases of hyperuricemia. MHOWO individuals had a 57% increased risk of hyperuricemia compared with MHNW individuals (OR, 1.57; 95% CI 1.15–2.13; *P* = .004), and the corresponding OR for MUHOWO was 2.45 (95% CI 1.87–3.19, *P* < .001). Notably, even MUNW individuals had the highest risk of developing hyperuricemia (OR, 2.58; 95% CI 1.81–3.67; *P* < .001). According to the results of blood uric acid value, no correlation was found between MHOWO individuals and blood uric acid value, but both MUNW and MUHOWO were positively correlated with blood uric acid value, which was consistent with the above results (Table 2).

**Table 2:** Multivariate-adjusted associations of different obesity phenotypes with serum uric acid levels and hyperuricemia.

	Serum	UA	Hyperuricemia				
Group	β	*P*	β	SE	Wald χ^2^	OR (95%CI)	*P*
MHNW	Ref					Ref	
MHOWO	0.013	0.46	0.45	0.16	8.10	1.57(1.15,2.13)	.004
MUNW	0.086	<0.001	0.95	0.18	27.67	2.58(1.81,3.67)	<.001
MUHOWO	0.095	<0.001	0.89	0.14	43.41	2.45(1.87,3.19)	<.001

Adjusted variables are BMI, gender, age, education, marital status, smoking, and drinking.

MHNW = metabolically healthy normal weight, MHOWO = metabolically healthy overweight/obesity, MUNW = metabolically unhealthy normal weight, MUHOWO = metabolically unhealthy overweight/obesity.

In addition, all metabolic obesity phenotypic transition features were examined during follow-up. Among participants with MHNW at baseline, only 10.7% converted to MHOWO. Among MHOWO participants, 21.2% converted to MHOWO, while the majority (52.6%) of MUHOWO did not convert throughout follow-up. Among the various phenotypes, 67.4% were not converted in the resurvey (Table 3).

**Table 3:** Transitions of different metabolic obesity phenotypes from baseline to follow-up review.

	Metabolic obesity phenotype at follow-up(%)				
Metabolic obesity phenotype at baseline	MHNW	MHO	MUNW	MUO	Total
MHNW	2534 (82.4)	328 (10.7)	137 (4.5)	76 (2.5)	3075 (100.0)
MHO	169 (13.9)	774 (63.8)	13 (1.0)	258 (21.2)	1214 (100.0)
MUNW	253 (52.2)	60 (12.3)	99 (20.4)	73 (15.1)	485 (100.0)
MUO	70 (5.4)	486 (37.8)	53 (4.1)	676 (52.6)	1285 (100.0)
Total	3026 (49.9)	1648 (27.2)	302 (5.0)	1083 (17.9)	6059 (100.0)

MHNW = metabolically healthy normal weight, MHO = metabolically healthy overweight/obesity, MUNW = metabolically unhealthy normal weight, MUO = metabolically unhealthy overweight/obesity.

At present, the MHOWO phenotype is the most controversial among various metabolic obesity phenotype transitions and disease associations. Therefore, this study mainly analyzed the association between the MHOWO phenotype transition and the risk of hyperuricemia. Both normal-weight and overweight and obese participants had an increased risk of hyperuricemia compared with participants whose MH remained stable, even in normal-weight participants when metabolic status remained unhealthy, the risk of hyperuricemia (OR = 3.09, 95%CI: 1.51–6.30, *P* < .01) was also higher than the risk of overweight and obese (OR = 2.02, 95%CI: 1.29–3.15, *P* < .01). For normal-weight subjects, the risk of hyperuricemia did not increase when transitioning from a metabolically healthy state to a metabolically unhealthy state (OR = 1.16, 95%CI: 0.78–3.34, *P* = .20). However, the risk of hyperuricemia was increased in overweight and obese individuals when they changed from a metabolically healthy state to a metabolically unhealthy state (OR = 2.09, 95%CI: 1.20–3.67, *P* = .01). Shifting from a metabolically unhealthy state to a healthy state in both normal-weight and overweight/obesity participants continued to increase the risk of hyperuricemia (see Table 4).

**Table 4:** Multivariate analysis of the association between different obesity phenotypes and the risk of hyperuricemia.

	Normal weight					Obesity/Overweight				
Type	β	SE	Wald χ^2^	OR (95%CI)	*P*	β	SE	Wald χ^2^	OR(95%CI)	*P*
MH to MH				Ref					Ref	
MH to MU	0.47	0.37	1.59	1.16(0.78,3.34)	.208	0.75	0.29	6.92	2.12(1.21,3. 27)	.009
MU to MU	1.16	0.36	10.14	3.09(1.51,6.30)	.001	0.71	0.23	9.71	2.03(1.30,3.17)	.002
MU to MH	0.87	0.27	10.34	2.31(1.36,3.92)	.001	0.50	0.25	3.90	1.65(1.00,2.70)	.048

MH = metabolically healthy, MU = metabolically unhealthy.

## 4. Discussion

This study is the first to identify the characteristics of metabolic obesity phenotype and its association with the risk of hyperuricemia in Chinese adults by using representative national population survey data. In Chinese adults, 21.2% of MHOWO metabolic obesity phenotypes were converted to MUHOWO during the 4-year follow-up period. Stable unhealthy metabolic state and transition from metabolically healthy state to unhealthy state will increase the risk of hyperuricemia. The risk of developing hyperuricemia in a stable unhealthy metabolic state remains higher than in a transition from metabolically healthy obesity (MHOWO) to unhealthy obesity (MUHOWO).

The results of this study suggest that individuals who remain metabolically unhealthy in a steady state have a higher risk of hyperuricemia than other categories of metabolically obese phenotypic shifts. Metabolic phenotypic shifts in both MHOWO and MUHOWO individuals increase the risk of hyperuricemia. In recent years, several studies have reported the association of the metabolic obesity phenotype with hyperuricemia. A cross-sectional study reported that the mean serum uric acid level in the MHOWO group was 41.87 μmol/L higher than that in the MHNW group, and the mean increase in the MUHOWO group was 63.18 μmol/L.^[11]^ A domestic study also came to a similar conclusion that hyperuricemia was positively correlated with the MHOWO, MUNW, and MUHOWO phenotypes, and this association was independent of gender and age.^[12]^ The results of this study also found that the serum uric acid value and the prevalence of hyperuricemia in the MHOWO, MUNW, and MUHOWO groups were higher than those in the MHNW group. Studies have pointed out that this association also has gender differences. Through Bayesian network inference, the probability of hyperuricemia in MHOWO men is 0.076, while that in MHOWO women reaches 0.124.^[13]^ However, there have been no reports of an association between metabolic obesity phenotypic shift and hyperuricemia. Previous domestic cohort studies on the association of metabolic obesity phenotype transition with cardiovascular disease also believed that for most Chinese adults, MHOWO is a transient state, stable metabolically unhealthy overweight or obese (MUHOWO) and metabolically unhealthy overweight or obese (MUHOWO). A transition from a healthy state to an unhealthy state is associated with a higher risk of developing observational disease than a stable healthy normal weight.^[14]^ The largest study with the longest follow-up period that addressed the metabolic phenotype reported by Eckel et al found that obesity was still a risk factor for cardiovascular disease even if metabolic health was maintained for a long time. Meanwhile, a large proportion of women with healthy metabolism have changed into unhealthy phenotypes over time, which is related to the increased risk of cardiovascular disease in all BMI categories.^[15]^ The underlying mechanism responsible for this difference is unclear. Available evidence suggests that insulin resistance may be involved, with metabolic disturbances more likely to lead to relative or absolute excess insulin secretion.^[16]^ Hyperinsulinemia reduces renal serum uric acid excretion, leading to subsequent hyperuricemia.^[17]^ But it is unclear whether metabolically healthy obese individuals can maintain insulin sensitivity throughout life, or whether metabolically healthy obesity simply represents a delayed onset of obesity-related insulin resistance.^[16]^

In addition, the elderly with MHNW and MHOWO phenotypes also have different degrees of transition to a metabolically unhealthy state, indicating that the metabolically healthy state is not a stable state. Even within a relatively short period of time, metabolically healthy older adults undergo a potential transition to a metabolically unhealthy phenotype. Previous studies have shown that approximately one-third to one-half of MHOWO individuals transition to a metabolically unhealthy phenotype.^[15,18]^ In the Nurses’ Health Study, Eckel et al found that 57%, 84%, and 94% of individuals with MHOWO at baseline converted to unhealthy status after 10, 20, and 30 years, respectively.^[15]^ The MHOWO conversion rates of the above studies were not consistent with the results of this study, possibly due to differences in the definition of metabolic health, the study population (ethnicity and age), and the length of follow-up. Emerging evidences showed that obese people with healthy metabolism might have a higher risk of all-cause mortality and cardiovascular events than obese people with healthy metabolism, but the risk was far lower than obese people with unhealthy metabolism. Therefore, obese people should actively carry out lifestyle intervention to promote the transformation from obesity with unhealthy metabolism to obesity with healthy metabolism, which could also reduce the risk of unhealthy health.^[19]^

Several previous studies have explored the effects of different components of the metabolic phenotype, including hyperlipidemia, hypertension, and hyperglycemia, on serum uric acid. Evidence from multiple studies supports the close relationship between hyperuricemia and hypercholesterolemia, hyperglycemia and hyperlipidemia.^[20,21]^ This also explains the findings of this study, which showed an increased risk of hyperuricemia for all metabolic phenotypes compared with the MHNW group. The increase in serum uric acid in obese individuals may be attributed to 2 factors: excess purine production due to excess energy and poor renal excretion.^[22]^

Although this study is a prospective cohort study representing a nationwide population, there are some limitations. First, the use of lipid-lowering, glucose-lowering, and blood pressure-lowering drugs was not considered at baseline in this study, and the use of these drugs may also have an effect on changes in the metabolic obesity phenotype during follow-up.^[23,24]^ Second, this study used BMI to define overweight/obesity without considering central obesity, which is an important predictor of metabolic disorders in some studies.^[25]^

Taken together, this study found that MHOWO would also increase the risk of hyperuricemia, with the highest risk of hyperuricemia in those who remained metabolically unhealthy but normal weight, both obese and normal weight individuals, even if metabolic status changes from unhealthy to healthy, there is still an increased risk of hyperuricemia. This study suggests that obesity is an important risk factor for hyperuricemia in Chinese adults even in the absence of metabolic syndrome. Therefore, close attention should be paid to the transition of metabolic health status over time, and individualized prevention strategies should be focused on metabolically unhealthy and obese individuals.

## Acknowledgments

The authors would like to thank the China Health and Retirement Longitudinal Survey (CHARLS) database for the support.

## Author contributions

Conception and design: Cheng Zhao. Administrative support: Cheng Zhao. Provision of study materials or patients: Cheng Zhao. Collection and assembly of data: Wenjing Zhao. Data analysis and interpretation: Wenjing Zhao. Manuscript writing: All authors. Final approval of manuscript: All authors.

**Conceptualization:** Cheng Zhao.

**Formal analysis:** Wenjing Zhao.

**Investigation:** Wenjing Zhao.

**Methodology:** Cheng Zhao.

**Resources:** Cheng Zhao.

**Software:** Wenjing Zhao.

**Supervision:** Cheng Zhao.
